# Linear Triquinane Sesquiterpenoids: Their Isolation, Structures, Biological Activities, and Chemical Synthesis

**DOI:** 10.3390/molecules23092095

**Published:** 2018-08-21

**Authors:** Yi Qiu, Wen-Jian Lan, Hou-Jin Li, Liu-Ping Chen

**Affiliations:** 1 School of Chemistry, Sun Yat-sen University, Guangzhou 510275, China; qiuyi0771@163.com; 2 School of Pharmaceutical Sciences, Sun Yat-sen University, Guangzhou 510006, China; lanwj@mail.sysu.edu.cn

**Keywords:** linear triquinane sesquiterpenoid, hirsutane, capnellene, isolation, structure, biological activity, synthesis

## Abstract

Linear triquinane sesquiterpenoids represent an important class of natural products. Most of these compounds were isolated from fungi, sponges, and soft corals, and many of them displayed a wide range of biological activities. On account of their structural diversity and complexity, linear triquinane sesquiterpenoids present new challenges for chemical structure identification and total synthesis. 118 linear triquinane sesquiterpenoids were classified into 8 types, named types I–VIII, based on the carbon skeleton and the position of carbon substituents. Their isolation, structure elucidations, biological activities, and chemical synthesis were reviewed. This paper cited 102 articles from 1947 to 2018.

## 1. Introduction

**Figure 1:**
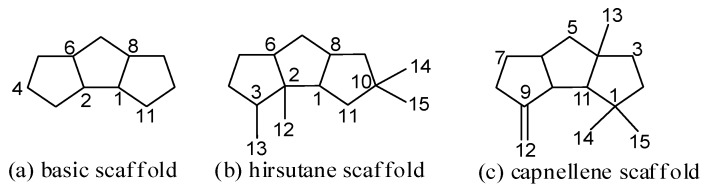
Skeleton of linear triquinane sesquiterpenoids.

**Figure 2:**
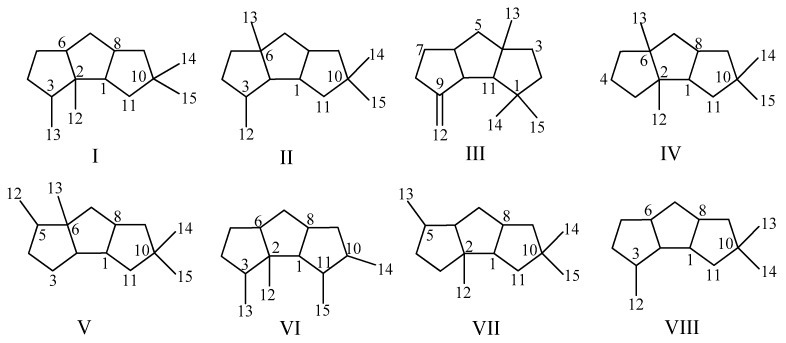
Structure types of linear triquinane sesquiterpenoids.

Sesquiterpenoids represent an important class of natural products. Among them, linear triquinane sesquiterpenoids have a basic skeleton 1*H*-cyclopenta[*a*]pentalene (Figure 1a) [1]. Since the first linear triquinane sesquiterpenoid, named hirsutic acid C obtained in 1947 [2,3], more than 100 linear triquinane sesquiterpenoids have been isolated, mainly from fungi, sponges, and soft corals. Many compounds displayed a wide range of biological activities, such as cytotoxic, antimicrobial, and anti-inflammatory activities [4,5,6,7]. Hirsutanes and capnellenes are the most common scaffolds of the linear triquinane sesquiterpenoids (Figure 1b,c) [8]. However, many compounds cannot be simply classified into these two types. Here, we categorize the linear triquinane sesquiterpenoids into eight types, types I–VIII, according to the carbon skeleton and the position of carbon substituents (Figure 2). This review gives an overview about the isolation, structure, biological activities, and chemical synthesis of linear triquinane sesquiterpenoids.

## 2. Isolation and Structure Elucidation

### 2.1. Type I

**Figure 3:**
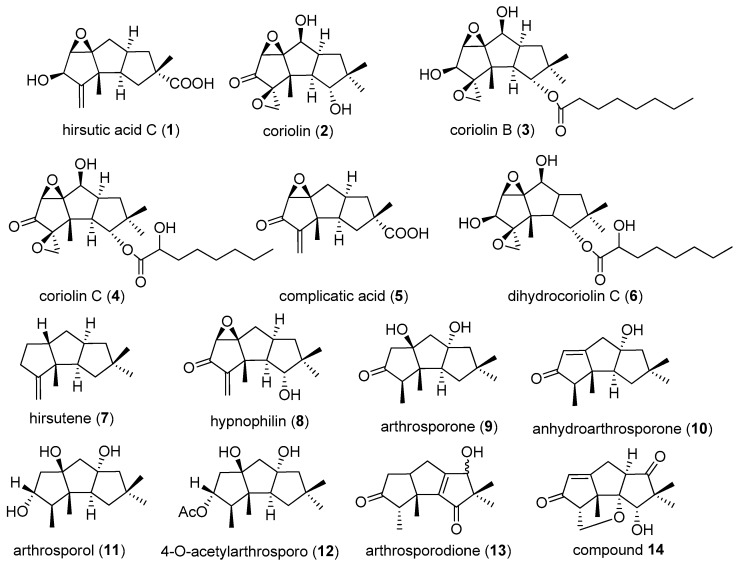

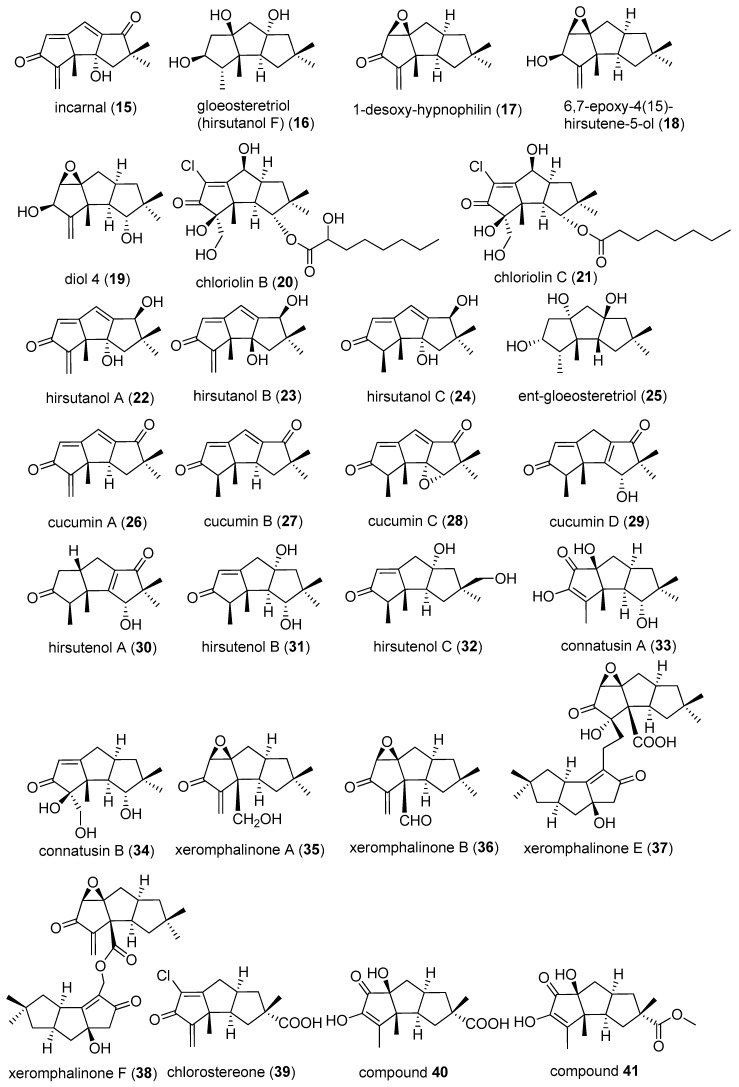

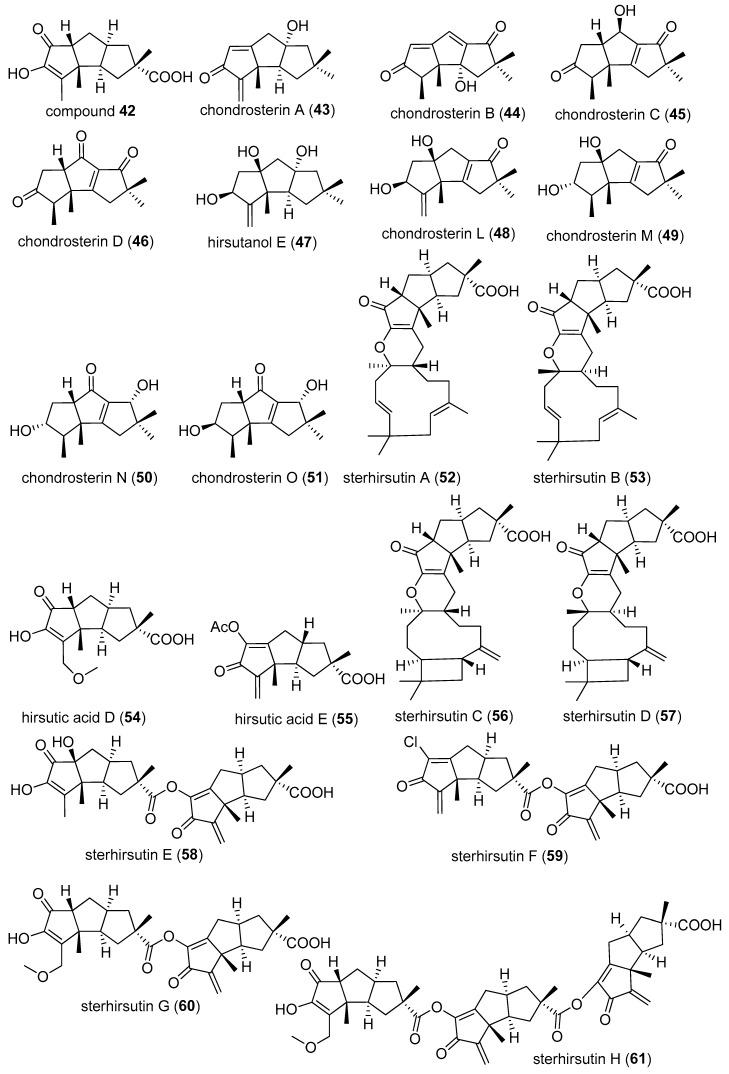

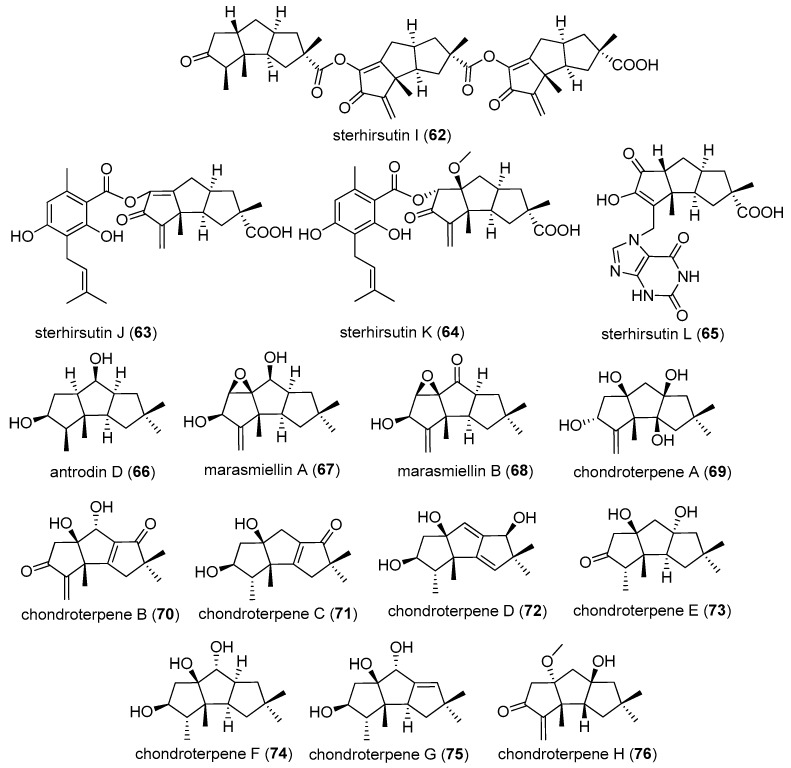
Structures of type I compounds.

There are four carbon substituents at C-2, 3, 10, 10 in type I compounds (Figure 3). A total of 76 compounds, constitute the largest classification of linear triquinane sesquiterpenoids. Hirsutic acid C (**1**), was the first linear triquinane sesquiterpenoids isolated from the cultures of *Stereum hirsutum* in 1947 [2,3]. The antibiotic coriolin (**2**), coriolin B (**3**), C (**4**), and hirsutene (**7**) were purified from the mycelia of basidiomycetes *Coriolus consors* [9,10,11]. However, the initial structures of coriolins were not exactly right, afterwards their structures and stereochemistry were revised by the same research group based on spectroscopic analysis, biogenetic consideration and chemical transformations, in 1971 [12]. The revised structures of coriolins showed that they were hirsutane-type sesquiterpenes. (−)-Complicatic acid (**5**) was first isolated from the fungus Stereum complicatum in 1973 [13]. 5-Dihydrocoriolin C (**6**) was obtained from *Coriolus consors* (ATCC 11574) [14].

Hypnophilin (**8**) was isolated from mycelial cultures of *Pleurotellus hypnophilus* (Berk.) Sacc. in 1981 [15,16]. Six new hirsutane metabolites, arthrosporone (**9**), anhydroarthrosporone (**10**), arthrosporol (**11**), 4-*O*-acetylarthrosporo (**12**), arthrosporodione (**13**), and compound **14** were obtained from fungus UAMH 4262 by Amouzou in 1989 [4]. Incarnal (**15**) was purified from the culture fluid of *Gloeostereum incarnatum* in 1991 [17]. In 1992, gloeosteretriol (**16**), also named hirsutanol F was obtained from the fluid fermentation products of *Gloeostereum incarnatum* [18]. From the culture extract of the fungus *Lentinus crinitus*, 1-desoxy-hypnophilin (**17**), and hypnophilin (**8**) were isolated as the most active compounds. In addition to these ketones, their corresponding alcohols 6,7-epoxy-4(15)-hirsutene-5-ol (**18**) and diol 4 (**19**) were obtained in 1994 [19].

Chloriolin B (**20**) and C (**21**) were purified from an unidentified fungus, which was separated from a marine sponge *Jaspis aff. johnstoni* [20].

Hirsutanols A–C (**22**–**24**) and ent­gloeosteretriol (**25**) were obtained from an unidentified fungus, separated from an Indo-Pacific sponge *Haliclona* sp. in 1998, and study showed that hirsutanol B was an epimer of hirsutanol A [5]. In 1998, from mycelial cultures of the agaric *Macrocystidia cucumis*, cucumis A–D (**26**–**29**) were found together with arthrosporone (**9**) [21]. Hirsutenols A–C (**30**–**32**) were isolated in 2002, from the culture broth of the mushroom *Stereum hirsutum* [22]. Connatusins A (**33**) and B (**34**) were isolated from the fungus *Lentinus connatus* BCC 8996, in 2005 [23].

Xeromphalinones A, B, E, F (**35**–**38**), were isolated from *Xeromphalina* sp. by Liermann et al. in 2010. At the same time, they isolated chlorostereone (**39**) from *Stereum* sp. [24]. In 2014, Ma and co-workers found three new sesquiterpenoids from the mycelia of *Stereum hirsutum*. Since they did not name these compounds at that time, we called them compounds **40**–**42** in proper order, successively [25].

Li and co-workers have studied *Chondrostereum* sp., which was separated from the soft coral *Sarcophyton tortuosum*, from the South China Sea. Their investigations found that the metabolites of *Chondrostereum* sp., were varied depending on the culture media. By altering the fermentation conditions, e.g., carbon and nitrogen source, *Chondrostereum* sp. can produce highly functionalized hirsutane type compounds with a surprising chemodiversity [26]. From the PDB (potato dextrose broth) medium, chondrosterins A–D (**43**–**46**) [26] were isolated, while hirsutanol E (**47**) [27], chondrosterins L–O (**48**–**51**) [28,29] were obtained from GPY (glucose-peptone-yeast) medium.

Aiming at finding new secondary metabolites, with bioactivities from fungi collected in the region of Tibet Plateau, a strain of *Stereum hirsutum* (L515) was isolated from its fruiting body. Sterhirsutins A (**52**) and B (**53**), hirsutic acids D (**54**) and E (**55**), were isolated from this strain’s solid-substrate fermentation culture in 2014 [30]. Additionally, in 2015, from its ethyl acetate extract, sterhirsutins C–L (**56**–**65**) were obtained [31]. Sterhirsutin L was the first sesquiterpene coupled with a xanthine moiety.

Antrodin D (**66**) together with coriolin (**2**), dihydrocoriolin C (**6**), and chloriolin B (**20**), were isolated from the fermentation of the basidiomycete *Antrodiella albocinnamomea* in 2015 [32].

Scale-up fermentation and chemical studies of basidiomycetes *Marasmiellus* sp. BCC 22389 led to the isolation and characterization of marasmiellins A (**67**) and B (**68**) in 2016. Marasmiellins are structurally, closely related to coriolins [33].

Separation of the active principles of the fermented broth of a fungus *Chondrostereum* sp. NTOU4196 which was isolated from the marine red alga *Pterocladiella capillacea*, resulted in the isolation of chondroterpenes A–H (**69**–**76**) in 2017 [34].

### 2.2. Type II

**Figure 4:**
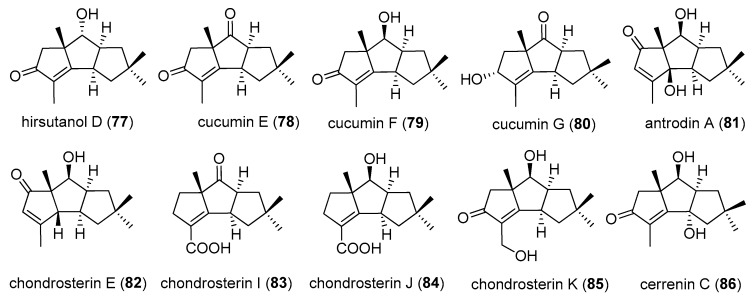
Structures of type II compounds.

There were 10 compounds belonging to type II, with four carbon substituents at C-3, 6, 10, 10 (Figure 4). 9 compounds a have carbonyl group.

Hirsutanol D (**77**) was produced from the terrestrial fungus *Corious consors,* cultured under both sea water and deionized water media, in 1998 [31]. Cucumins E–G (**78**–**80**) were isolated from mycelial extract of the agaric *Macrocystidia cucumis*, in 1998 [21]. From the fermentation of basidiomycete *Antrodiella albocinnamomea*, antrodin A (**81**) was obtained, in 2015 [32]. Chondrosterins E, I–K (**82**–**85**) were purified from the marine fungus *Chondrostereum* sp. [26,28,35]. Cerrenin C (**86**) was obtained from the broth extract of *Cerrena* sp. A593 [36].

### 2.3. Type III

**Figure 5:**
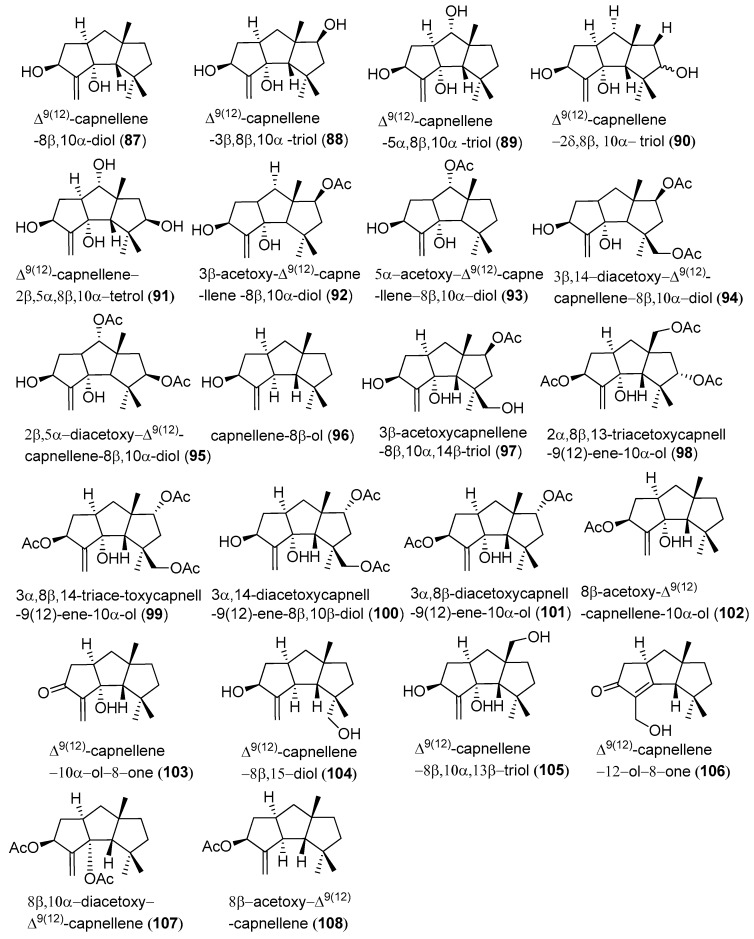
Structures of type III compounds.

In type III compounds, there are four carbon substituents in C-1, 1, 4, 9 (Figure 5). The most notable feature of these compounds is they contain an off-ring carbon-carbon double bond at C-9.

∆^9(12)^-capnellene-8β,10α-diol (**87**), ∆^9(12)^-capnellene-3β, 8β, 10α-triol (**88**), ∆^9(12)^-capnellene-5α,8β,10α-triol (**89**), and ∆^9(12)^-capnellene-2ξ,8β,10α-triol (**90**), were obtained from the soft coral *Capnella imbricata*, by Sheikh et al. These compounds were the first members of a new sesquiterpene class, which was named capnellane [37]. ∆^9(12)^-Capnellene-2β,5α,8β,10α-tetrol (**91**), has been isolated from the alcyonarian *Capnella imbricate,* by Kaisin et al. Its structure and relative configuration was determined by X-ray diffraction analysis, in 1979 [38]. In 1985, Kaisin and co-workers implemented the isolation of other eight acetylated capnellenes from acetone extraction of fresh colonies of *C. imbricate*. Four of these compounds were named as 3β-acetoxy-∆^9(12)^-capnellene-8β,10α-diol (**92**), 5α-acetoxy-∆^9(12)^-capnellene-8β,10α-diol (**93**), 3β,14-diacetoxy-∆^9(12)^-capnellene-8β,10α-diol (**94**), and 2β,5α-diacetoxy-∆^9(12)^-capnellene-8β,10α-diol (**95**) [39].

In 1998, capnellene-8β-ol (**96**) and 3β-acetoxycapnellene-8β, 10α,14β-triol (**97**) were isolated from *Capnella imbricata*, collected from Mayu Island, Molucca Sea, Indonesia, in 1996 [40]. 2α,8β,13-Triacetoxycapnell-9(12)-ene-10α-ol (**98**), 3α,8β,14-triace-toxycapnell-9(12)-ene-10α-ol (**99**), 3α,14-diacetoxycapnell-9(12)-ene-8β,10α-diol (**100**), and 3α,8β-diacetoxycapnell-9(12)-ene-10α-ol (**101**), were obtained from the soft coral *Dendronephthya rubeola*, in 2007 [6]. 

From the acetone/methylene chloride extracts of the Formosan soft coral *Capnella imbricate*, 8β-acetoxy-∆^9(12)^-capnellene-10α-ol (**102**), ∆^9(12)^-capnellene-10α-ol-8-one (**103**), ∆^9(12)^-capnellene-8β,15-diol (**104**), ∆^9(12)^-capnellene-8β,10α, 13β-triol (**105**), Δ^9(10)^-capnellene-12-ol-8-one (**106**), 8β,10α-diacetoxy-∆^9(12)^-capnellene (**107**), and 8β-acetoxy-∆^9(12)^-capnellene (**108**), were purified in 2008 [7].

### 2.4. Type IV, V, VI, VII and VIII

**Figure 6:**
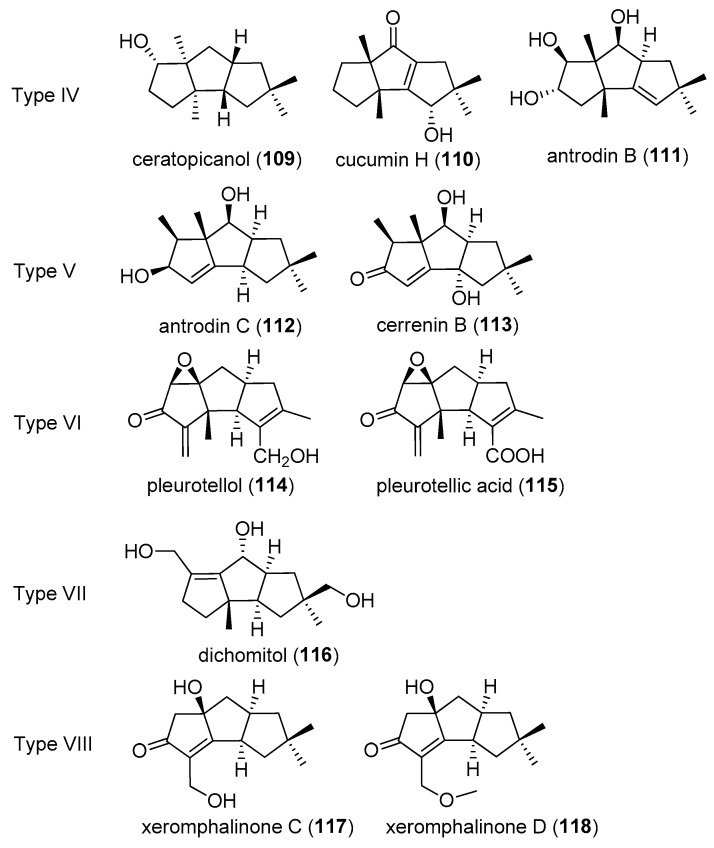
Structures of type VI–VIII compounds.

The number of type IV*–*VIII compounds (Figure 6), is relatively small. It is worth mentioning that type VIII compounds, have one less carbon substituent on the skeleton than other compounds.

Type IV: Ceratopicanol (**109**) was isolated from the fungus *Ceratosystis piceae,* in 1988. *Ceratosystis piceae* (Munch) Bakshi, is a sapwood staining ascomycete, occurring on coniferous loga and lumber [41]. Cucumin H (**110**) was found in 1998, from mycelial cultures of the agaric *Macrocystidia cucumis* [21]. From fermentation of *Antrodiella albocinnamomea*, antrodin B (**111**) was isolated in 2015 [32].

Type V: Antrodin C (**112**), was obtained from fermentation of *Antrodiella albocinnamomea,* in 2015 [32]. Cerrenin B (**113**) was isolated from the broth extract of *Cerrena* sp. A593, in 2018 [36].

Type VI: Pleurotellic acid (**114**) and pleurotellol (**115**) were isolated from mycelial cultures of *Pleurotellus hypnophilus* (Berk.), in 1986 [15,16].

Type VII: Dichomitol (**116**), was purified from mycelial solid cultures of *Dichomitus squalens*, which was a commonly found white-rot basidiomycete fungus, in 2004 [42].

Type VIII: Xeromphalinones C (**117**) and D (**118**), were obtained from *Xeromphalina* sp. by Liermann et al., in 2010 [24].

From the perspective of source, linear triquinane sesquiterpenoids can be isolated from both marine and terrestrial organisms, however, the proportion of terrestrial species is relatively larger. It is worthwhile to note that, there are several highly productive biological species, such as the mushroom *Stereum hirsutum*, the soft coral *Capnella imbricata,* and marine fungus *Chondrostereum* sp.

Structurally, there are 76 compounds belonging to type I, accounting for the majority of these 118 compounds, and 22 compounds constitute type III. In fact, it was most common to categorize linear triquinanes, according to their ring scaffold with the hirsutanes and capnellenes. Type I compounds, exactly belong to hirsutanes, while type III belong to capnellenes. 

As for functional groups, the epoxy bond is easy to form at C-6, 7 positions and the majority of carbonyls are formed at C-4, C-7, and C-9 positions, with a minority of carbonyls formed at C-5. As regards the hydroxyl group, except for C-10, it is possible to form at the other 10 positions.

Additionally, Kutateladze and Kuznetsov used the relatively fast parametric/DFT hybrid computational method DU8+, to analyze the reported NMR data for 90+ natural triquinanes in 2017, which resulted in requiring structure correction of 13 compounds, including 10 linear triquinane sesquiterpenoids, noted in this review [43]. However, this review still used the structures from the original literatures, because only the calculations were not convincing enough.

## 3. Biological Activity

### 3.1. Cytotoxicity

Cytotoxicity was a kind of common biological activity reported for linear triquinane sesquiterpenoids, usually expressed as inhibitory concentration (IC_50_), 50% of the effective dose (ED_50_), cytotoxic concentration (CC_50_), lethal concentration (LC_50_), or growth inhibition (GI_50)_, this value emphasizes the correction for the cell count at time zero) [1]. Among the 118 linear triquinane sesquiterpenoids, many compounds exhibited activities against cancer cell lines (Table 1).

Coriolin B (**3**) inhibited human breast cancer cell T-47D and CNS cancer cell line SNB-75, with the IC_50_ values of 0.7 and 0.5 μM, respectively [20].

Toxicity assays were carried out with L929 mouse fibroblasts cells. The IC_50_ values were 2.4 μg/mL for 1-desoxy-hypnophilin (**17**) and 0.9 μg/mL for 6,7-epoxy-4(15)-hirsutene-5-ol (**18**) [19]. 

Cucumins A–C (**26**–**28**) were cytotoxic, with IC_100_ range from 0.5 to 1 mg/mL for L1210 cells [21]. The cytotoxicities (IC_50_ value) of xeromphalinones A (**35**), B (**36**), F (**38**) and chlorostereone (**39**) against Jurkat cells, ranged from 1 to 5 μg/mL (2 to 20 μM). For xeromphalinones C–E (**117**–**118**, **37**), the IC_50_ values were above 50 μg/mL [24].

Compound **40** inhibited the growth of HepG2 cells, with IC_50_ value of 24.41 ± 1.86 μM [25]. Chondrosterin A (**43**) showed significant cytotoxicity against cancer lines A549, CNE2, and LoVo, with IC_50_ values of 2.45, 4.95, and 5.47 Μm, respectively, whilst chondrosterins B–E (**44**–**46**, **48**) were apparently inactive (IC_50_ > 200 μM), in the assay developed by Li [26]. Additionally, in Hsiao and co-workers’ investigation, chondrosterins A (**43**) and B (**44**) and chondroterpene H (**76**), showed potent cytotoxic activities on BV-2 cells, at a concentration of 20 μM [34]. Chondrosterin J (**84**) showed potent cytotoxic activities against the cancer cell lines CNE1 and CNE2, with the IC_50_ values of 1.32 and 0.56 μM, respectively. By comparison, chondrosterin J (**84**) showed stronger cytotoxic activities than chondrosterin A (**43**) (CNE2: 4.95 μM), hirsutanol A (**22**) (CNE1:10.08 μM; CNE2: 12.72 μM), and incarnal (**15**) (CNE1: 34.13 μM; CNE2: 24.87 μM). In contrast, chondrosterin I (**83**) was inactive (IC_50_ values > 200 μM) [35]. Yang and co-workers have investigated the molecular mechanisms of the antitumor activity of hirsutanol A, with the result that hirsutanol A inhibited tumor growth, through triggering ROS production and apoptosis [44]. Chondrosterins K–M (**85**, **48**–**49**) showed cytotoxicities against seven cancer cell lines CNE1, CNE2, HONE1, SUNE1, A549, GLC82, and HL7702, in vitro [28].

Sterhirsutins A–B (**52**–**53**) and hirsutic acid D–E (**54**–**55**) showed cytotoxicity against K562 and HCT116 cell lines. They exhibited cytotoxicity against K562 cells, with the IC_50_ values of 12.97, 16.29, 6.93, and 30.52 μg/mL, respectively. For the HCT116 cell line, they showed cytotoxicity with the IC_50_ values of 10.74, 16.35, 25.43, and 24.17 μg/mL [30]. Sterhirsutins E–G (**58**–**60**) and I (**62**) also showed antiproliferative activity against K562 and HCT116 cancer cells, with IC_50_ values ranging from 6 to 20 μM.

Compound **87** was cytotoxic toward human promyelocytic leukemia cell line HL-60, human erythroleukaemia cell line K562, human renal leiomyoblastoma cell line G402, human breast adenocarcinoma cell line MCF-7, human colon-cancer cell line HT 115, and human ovarian cancer cell line A2780, with IC_50_ values in the range of 0.7–93 μM, and the greatest activity was displayed against K562 at 0.7 μM. Compound **95** showed some selectivity, for the renal leiomyoblastoma and ovarian cancer cell lines. Compound **96** was effective against the K562, as well as HL-60 [40].

Compounds **98**–**101** showed weak cytotoxic activities against the human cervix carcinoma cell line HeLa, but they also showed weak or moderate antiproliferative effects, against the cell lines K562 and murine fibroblast cell line L-929 [6].

Compounds **87** and **102** exhibited strong activity against HeLa and L-929. Compounds **87**, **101**, **102** showed similar anti-proliferative activities against K562. The anti-proliferative and cytotoxic activity of compound **84** against L-929 and HeLa cells, is 5.7 and 3.8 times lower than doxorubicin, which was used in the cancer therapy for the treatment of lymphoma, carcinoma, sarcoma, and leukemia [6].

### 3.2. Antimicrobial Activity

Coriolin (**2**) showed inhibitory effect on the growth of Gram-positive bacteria at 6.25–12.5 mcg/mL, some of Gram-negative bacteria at 50–100 mcg/mL [9].

(−)-Complicatic acid (**5**) was against a range of Gram-positive and Gram-negative bacteria [42]. Compounds **9**–**11** displayed weak antifungal activity [4]. Incarnal (**15**) inhibited the growth of Gram-positive bacteria at 6.25–12.5 µg/mL, while it had no antibacterial activity against Gram-negative bacteria [17].

Compounds **17** had stronger antimicrobial activity than **18** (Table 2) [19]. Compounds **22** and **25**, were found to be anti-microbial (*Bacillus subtilus*) active [5]. Hirsutenols A–C (**30**–**32**) showed moderate antibacterial activity against *Escherichia coli* [22].

### 3.3. Other Bioactivity

Compounds **22**, **43**–**44**, **69**–**70**, and **76**, inhibited lipopolysaccharide (LPS)-induced NO production in BV-2 microglial cells, at a concentration of 20 μM. Compounds **22** (5–20 μM) and **69**, had less effects on cell viability. At the same concentration, **24** and **70**–**75** had little effect on viabilities. Only **22**, with an α-exomethylene carbonyl moiety, was more potent than **24**, on inducing cellular toxicity. Compounds **43** and **76**, reduced cellular viability, more than **22**. Compounds **43**, **44** (20 μM), and **76**, all decreased the NO levels to the resting condition. These results suggested that cell death, predominantly caused decreasing NO production. However, the decreased NO levels by **22** (5 μM) and **69**, were dependent on inhibition of NO production instead of cell death [34] (Table 3 and Table 4).

Compound **40** exhibited NO inhibitory effect, on the LPS-induced macrophages, with the IC_50_ value of 15.44 ± 1.07 μM [25]. 

Sterhirsutin G (**60**) showed inhibitory activity on the activation of IFNβ promoter at a dose of 10 μM in HEK293 cells, whereas other compounds exhibited no apparent inhibitory effect on the IFNβ promoter activation, under the same experimental conditions. Furthermore, **60** dramatically reduced the SeV-induced mRNA level of IL6, RANTES, and IFNB1. Compared with **60**, sterhirsutin E (**58**) showed no inhibitory activity on the IFNβ promoter activation. These findings implied that sterhirsutin G, has the ability to inhibit the RLRs-mediated antiviral signaling in cells, and was potent to be a leading compound in the treatment of autoimmune diseases [31].

Sterhirsutin K (**64**) induced autophagy in HeLa cells, and its autophagy-inducing effect was evaluated via quantification of the number of GFP-LC3 dots in cells. Sterhirsutins A–L (**52**, **53**, **56**–**65**) and hirsutic acids D (**54**) and E (**55**), were screened using GFP-LC3 stable HeLa cells. As a result, **64** was found to exhibit strong autophagy-inducing activity at a dose of 50 μM. In comparison with hirsutic acid E (**55**), sterhirsutin J (**63**) and K (**64**) showed cytotoxicity rather than autophagy-inducing effect, on GFP-LC3 stable HeLa cells, at the concentration of 50 μM (inhibition rate = 100%). The double bond between C-6 and C-7 in **55** and **63** increased cytotoxicity, but significantly decreased the autophagy-inducing activity [31].

Compound **86** showed a specific inhibition (77%) of the Myc/Max interaction in yeast, and a lower inhibition of the Tax–CREB interaction (27%) and Myc–Miz-1 interaction (34%). This specific inhibition of Myc–Max interaction, is well associated with its antiproliferative effect against the L-929 cell line [6].

Compounds **87**, **102**, and **103**, significantly reduced the levels of the iNOS protein at concentrations of 10 μM, in comparison with control cells stimulated with LPS. **87** and **102** dramatically reduced the levels of the COX-2 protein at a dose of 10 μM, compared with control cells stimulated with LPS [7]. 

The anti-inflammatory effects of compounds **87**, **96**, and **103**–**109** were tested using LPS-stimulated cells. Stimulation of RAW 264.7 cells with LPS led to up-regulation of the pro-inflammatory iNOS and COX-2 proteins [7].

As a summary, among these 118 compounds, there were 56 compounds showing biological activities. Hirsutanes and capnellenes are major active compounds. They have cytotoxic, antimicrobial, anti-inflammatory activities, and so on. Previous reports revealed that the compounds with a α-methylidene oxo group, such as hypnophilin (**8**) and desoxyhypnophilin (**17**), possessed stronger antimicrobial or cytotoxic activities [26]. These structures and bioactivities are a source of inspiration, for synthetic chemists and pharmaceutist, to develop innovative methodologies in the field of medicine chemistry.

## 4. Chemical Synthesis

Linear triquinane sesquiterpenoids, have attracted a lot of interest among synthetic chemists in recent decades, owing to their premium structures and promising biological activities [45]. Their skeletal and functional diversities, also pose a considerable synthetic challenge [46].

### 4.1. Hirsutane Type

In the early 1970s, chemists began to synthesize hirsutane type compounds, in a variety of ways. 

**Figure 7:**
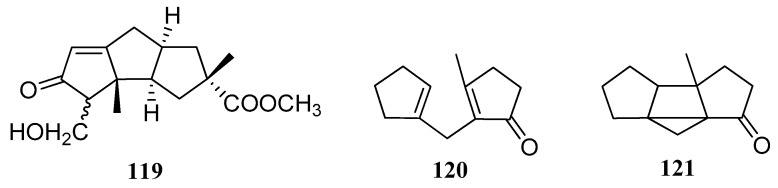
Structures of compounds **119**–**121**.

The total synthesis of hirsutic acid C (**1**), was achieved via the suitably functionalized skeleton compound **119** (Figure 7). In 1974, Hisanobu Hashimoto and co-workers reported a stereospecific conversion of 3α-hydroxymethylene ketone (compound **119**) to racemic hirsutic acid [47,48]. In 1978, Kueh and co-workers [49] reported the synthesis of the hirsutane carbon skeleton. They found that the photochemical intramolecular [2 + 2] cycloaddition of dicyclopent-l-enylmethanes (**120**), followed by in situation of methanol to the strained cyclopropane ring in the presumed intermediate bicycle[2.1.0]pentanes (**121**), led to an easy synthesis of the ring system.

**Scheme 1:**
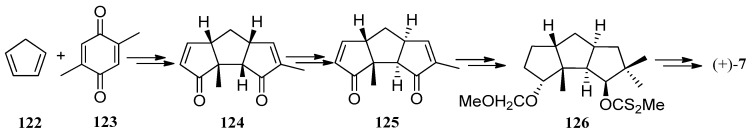
The simple expedient synthesis of (+)-hirsutene ((+)-**7**) via a novel photo-thermal metathetic sequence.

In 1981, Mehta and co-workers developed a simple expedient synthesis of (+)-hirsutene ((+)-**7**) via a novel photo-thermal metathetic sequence (Scheme 1). There were three distinctive features in this method: (1) the *cis*-syn-*cis*-C_13_-tricyclopentanoid frame (**124**) was efficiently obtained from cyclopentadiene and 2,5-dimethyl-p-benzoquinone, in three high-yielding steps employing only heat and light as the reagents; (2) compound **124** with the requisite cis-anti-cis system (**125**), was in ready and remarkable thermal equilibration; and (3) generation of abundant functionality on the tricyclopentanoid frame permits the synthesis of the higher oxygenated members of the hirsutane type compounds [50].

In 1984, Dawson and co-workers reported a simple stereocontrolled sequence to synthesize hirsutene from dicyclopentadiene which contained ring expansion by ketonization of a cyclopropoxide and skeletal rearrangement, through a β-enolate [51]. A primary feature of this synthesis, was that the stereochemistry at three of the four chiral centers was established essentially at the beginning of the sequence, and then the β-enolate rearrangement specifically generated the correct configuration at the fourth center, exclusively.

**Scheme 2:**
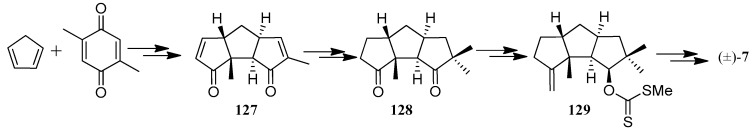
The total synthesis of (±)-hirsutene ((±)-**7**).

**Scheme 3:**
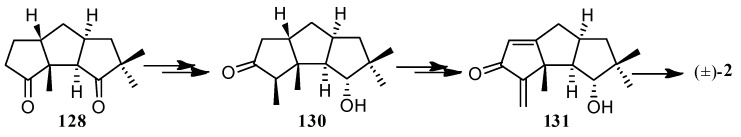
The total synthesis of (±)-coriolin ((±)-**2**).

In 1986, Mehta and co-workers [52] developed a simple method for triquinane natural products, based on photo-thermal olefin metathesis of readily available Diels-Alder adducts. They also applied this approach to the total synthesis of (±)-hirsutene ((±)-**7**) (10 steps, Scheme 2) and (±)-coriolin ((±)-**2**) (17 steps, Scheme 3).

**Scheme 4:**
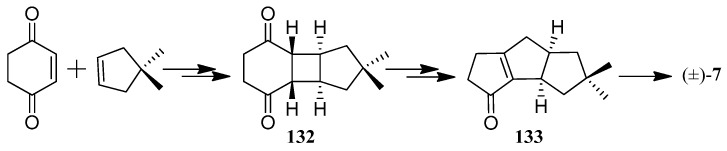
The short synthesis of (±)-**7**.

The trimethylsilyl iodide-mediated rearrangement of a bicyclo[4.2.0]octane-2,5-dione to a bicyclo[3.3.0]-oct-1(6)-en-2-one, was achieved by Oda and co-workers, for a short synthesis of (±)-hirsutene ((±)-**7**) (Scheme 4) [53].

**Scheme 5:**
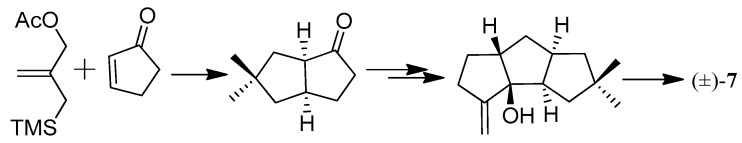
The synthesis of (±)-**7** utilizing photochemical reduction.

The photochemical reduction of a δ, ε-unsaturated ketone, was a key step to synthesize (±)-hirsutene ((±)-**7**), reported by Cossy and co-workers (Scheme 5) [54].

**Scheme 6:**
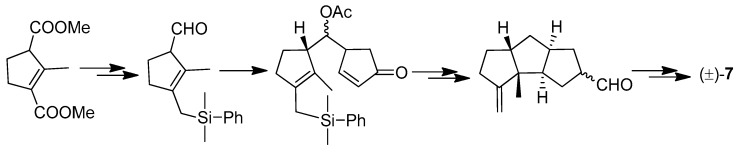
The synthesis of (±)-**7** via the intramolecular addition.

Majetich and co-workers achieved a synthesis of (±)-hirsutene ((±)-**7**), via the intramolecular addition of two functionalized cyclopentane rings, linked by a one-carbon tether (Scheme 6) [55].

**Scheme 7:**
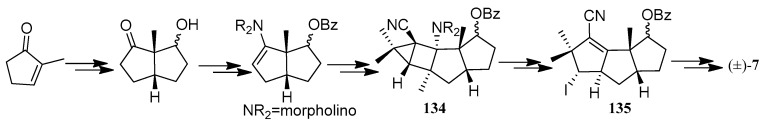
The synthesis of (±)-**7** via the region and stereospecific cleavage.

In Franck-Neumann and co-workers’ approach to (±)-hirsutene ((±)-**7**) (Scheme 7), the key strategy was the region and stereospecific cleavage (**134**→**135**) of the central bond in the resulting bicyclo[2.1.0]-pentane moiety [56,57,58].

**Scheme 8:**
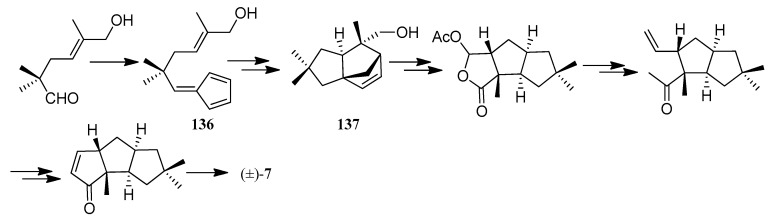
The highly efficient synthesis of (±)-**7**.

Sternbach and Ensinger achieved a highly efficient 20-step synthesis of (±)-hirsutene ((±)-**7**) (Scheme 8) [59]. An intramolecular Diels-Alder reaction of the substituted cyclopentadiene (**136**), which contains all but two of the carbon atoms found in hirsutene, serves as the key step in this sequence.

**Scheme 9:**
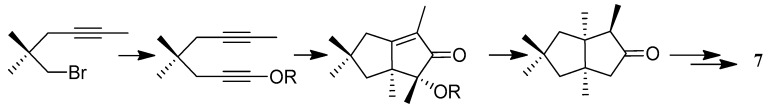
The enantioselective formal synthesis of hirsutene (**7**).

Castro and co-workers accomplished an enantioselective formal synthesis of hirsutene (**7**), employing an asymmetric Pauson-Khand bicyclization [60] (Scheme 9).

**Scheme 10:**
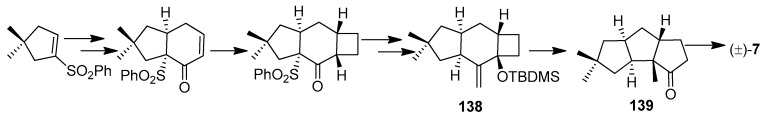
The total synthesis of (±)-**7** via a twofold cycloaddition.

Paquette and co-workers have achieved a total synthesis of (±)-hirsutene ((±)-**7**) in 9 steps and in 14% overall yield via a twofold cycloadditive scheme, utilizing an iodine catalyzed rearrangement of the methylenetricyclo[6.3.0.0^3,6^]undecane derivative (**138**) into the norketone (**139**), a commonly used precursor of hirsutene (**7**) [61] (Scheme 10).

**Scheme 11:**
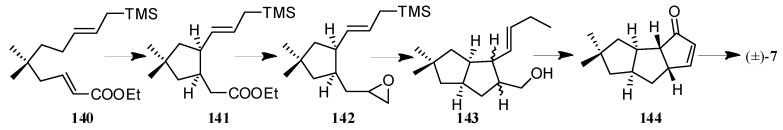
The formal synthesis of (±)-**7** via an intramolecular ene reaction and a titanium catalyzed epoxy-allylsilane cyclization.

In the formal synthesis of (±)-hirsutene ((±)-**7**), by Sarkar and co-workers [62,63,64,65] (Scheme 11), two key cyclopentannulation protocols, leading to the diquinane **141**, were an efficient intramolecular ene reaction (**140**→**141**) and a titanium catalyzed epoxy-allylsilane cyclization (**142**→**143**). Then, **143** was further transformed to the **144**, via the classical aldol condensation.

**Scheme 12:**
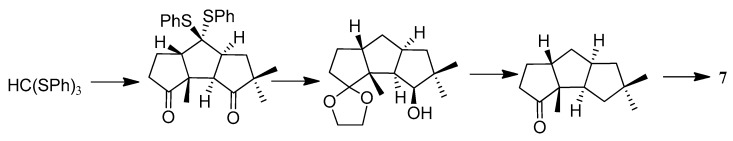
The total synthesis of (±)-**7** via a novel one-flask [2+1+2] cyclopentannulation reaction.

Keith and co-workers [66] achieved a highly efficient total synthesis of (±)-hirsutene ((±)-**7**), by using dilithiomethane equivalent in a novel one-flask [2 + 1 + 2] cyclopentannulation reaction (Scheme 12).

**Scheme 13:**
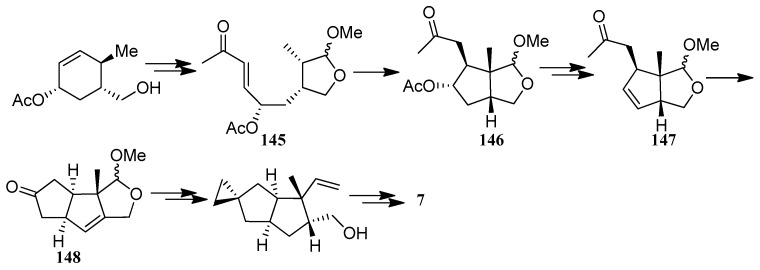
The synthesis of **7** utilizing an acid catalyzed intramolecular conjugate addition and a Pd^2+^-promoted highly stereocontrolled cyclization.

Toyota and co-workers developed a new, highly diastereocontrolled approach, for the synthesis of hirsutene (**7**), in which the key step utilized an acid catalyzed intramolecular conjugate addition (**145**→**146**), and a Pd^2+^-promoted highly stereocontrolled cyclization (**147**→**148**) [67] (Scheme 13).

**Scheme 14:**
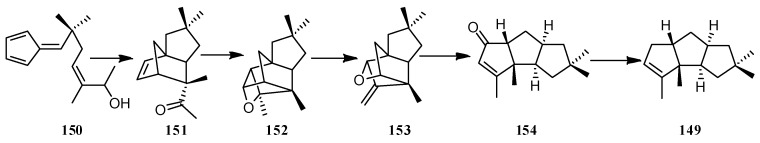
The stereocontrolled synthesis of (±)-endo-hirsutene (**149**) via intramolecular Diels-Alder, Paterno-Buchi reactions and a reductive fragmentation.

An efficient stereocontrolled synthesis of (±)-endo-hirsutene (**149**) was achieved, in which the key steps included intramolecular Diels-Alder, Paterno-Buchi reactions, and a reductive fragmentation [68]. (±)-Endo-hirsutene (**149**), earlier converted to (±)-hirsutene ((±)-**7**), was furnished from the deoxygenation of triquinane **154** (Scheme 14).

**Scheme 15:**
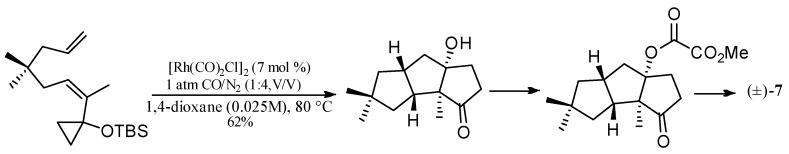
The synthesis of (±)-**7**.

**Scheme 16:**
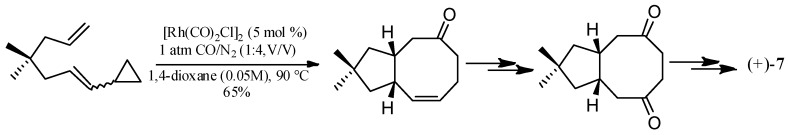
The synthesis of (+)-hirsutene ((+)-**7**).

**Scheme 17:**
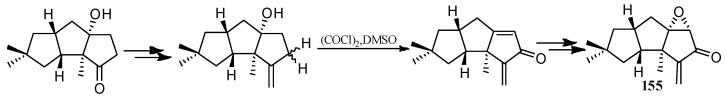
The synthesis of 1-desoxyhypnophilin (**155**).

**Scheme 18:**
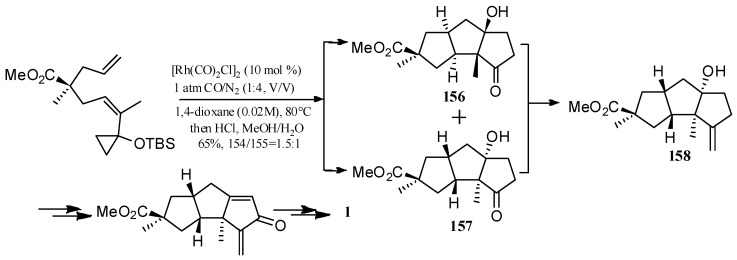
The synthesis of hirsutic acid C (**1**).

Wang and Yu described a rhodium-catalyzed [5+2+1] cycloaddition of ene-vinylcyclopropanes and CO, and applied this method to synthesize (±)-hirsutene ((±)-**7**) (Scheme 15), (+)-hirsutene ((+)-**7**) (Scheme 16), 1-desoxyhypnophilin (**155**) (Scheme 17), and hirsutic acid C (**1**) (Scheme 18) [69].

**Scheme 19:**
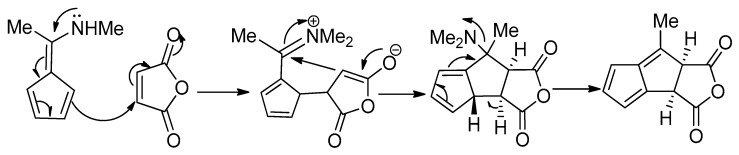
The facile synthesis of the hirsutane framework.

A facile synthesis of the hirsutane framework was reported in 2002 [58]. This method was via a novel [6 + 2] cycloaddition of fulvenes with alkenes, as shown in Scheme 19.

**Scheme 20:**
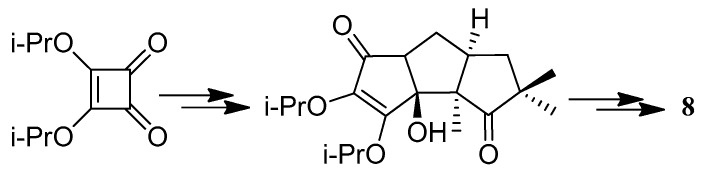
The synthesis of hypnophilin (**8**) via a combination of chlorination, reduction, dehydration and oxidation maneuvers.

In 2002, Geng and co-workers presented a direct 10-step route from a squarate ester to hypnophilin (**8**) [70]. The access route involved a combination of chlorination, reduction, dehydration, and oxidation maneuvers in the proper sequence (Scheme 20).

**Scheme 21:**
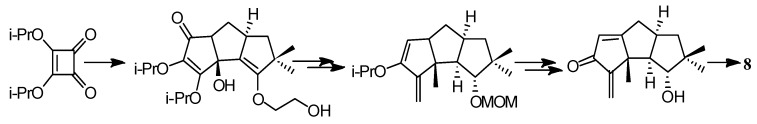
The conversion of diisopropyl squarate to **8.**

**Scheme 22:**
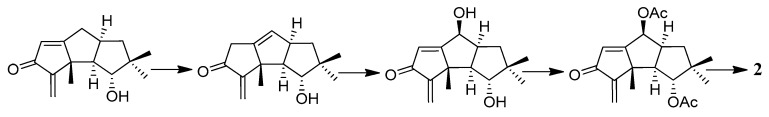
The formal synthesis of racemic coriolin (**2**).

**Scheme 23:**
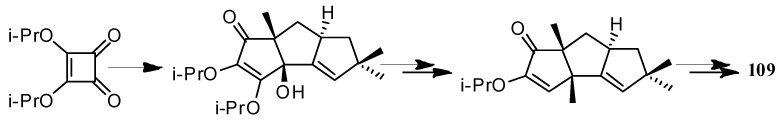
The synthesis of ceratopicanol (**109**).

The first application of the squarate ester cascade to natural products synthesis, was realized by Paquette and Geng, in 2002 [71]. Only 10 laboratory steps were needed to achieve the conversion of diisopropyl squarate to hypnophilin (**8**) (Scheme 21). A penultimate precursor to **8**, has previously been transformed to **2** (Scheme 22), thereby achieving a formal synthesis of racemic **2**. A rather different strategy was employed to synthesize ceratopicanol (**109**) (Scheme 23).

**Scheme 24:**
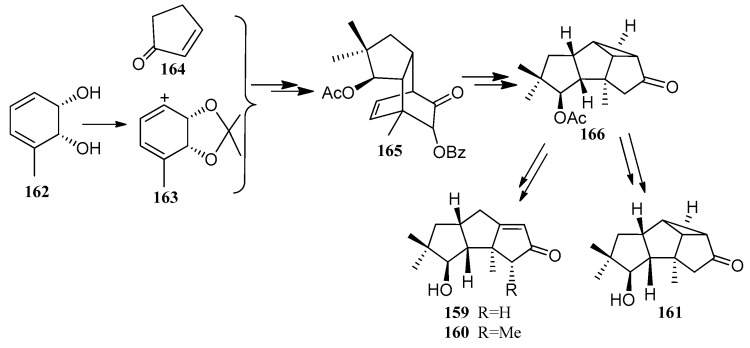
The synthesis of compound **159**, an advanced intermediate of both compounds (−)-**8** and (−)-**2** and the synthesis of the isomeric tetracyclic system **161**, a late-stage precursor to (−)-**2**.

In 2013, Bon and co-workers accomplished the formal total syntheses of (−)-hypnophilin ((−)-**8**) and (−)-coriolin ((−)-**2**) [72]. In particular, they reported the synthesis of compound **159**, an advanced intermediate related with previously reported syntheses of both compounds (−)-**8** and (−)-**2**. They attempted to prepare its methylated congener **160**, and the generation of the isomeric tetracyclic system **161**, representing a late-stage precursor to (−)-**2** (Scheme 24).

**Scheme 25:**
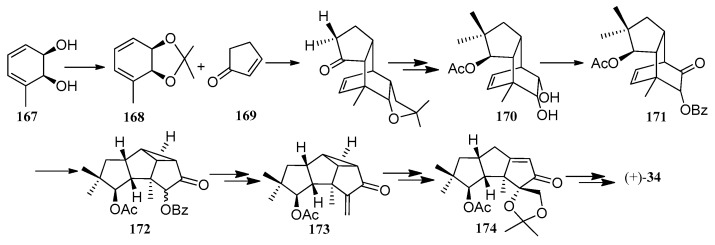
The synthesis of (+)-connatusin B ((+)-**34**) via reductive cleavage of the three-membered ring within this framework and kinds of functional group interconversions.

**Scheme 26:**
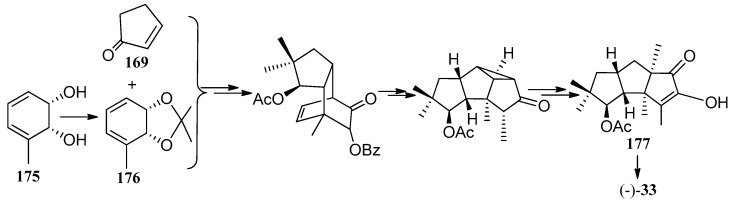
The synthesis of (−)-connatusin A ((−)-**33**).

Bon and co-workers showed great interest in the synthesis of the linear triquinane sesquiterpenoids. In 2010, they reported a chemoenzymatic total synthesis of (+)-connatusin B ((+)-**34**) from toluene [73]. In this synthesis, the starting material employed the *cis*-1,2-dihydrocatechol (**167**), which was acquired in enantiomerically pure via the enzymatic dihydroxylation of toluene. Diels-Alder cycloaddition and oxa-di-π-methane rearrangement reactions, represented the key steps in the reaction sequence, leading to the cyclopropane ring-fused linear triquinane (**172**). Reductive cleavage of the three-membered ring within this framework and types of functional group interconversions, then provided (+)-connatusin B ((+)-**34**) (Scheme 25). In 2011, the researchers applied this work to the synthesis of (−)-connatusin A ((−)-**33**). Notably, the final step of this sequence required cleaving the acetate residue within compound **177**, to reveal the corresponding alcohol, and they could not secure good yields of the final product. Eventually, after considerable experimentation, they found that the use of acidified DOWEX-50WX-100 resin in 5:1 *v*/*v* methanol at 65 °C for 18 h, showed the most effective result and provided a crystalline sample of (−)-connatusin A ((−)-**33**) in 84% yield (Scheme 26) [74].

### 4.2. Capnellene Type

**Figure 8:**
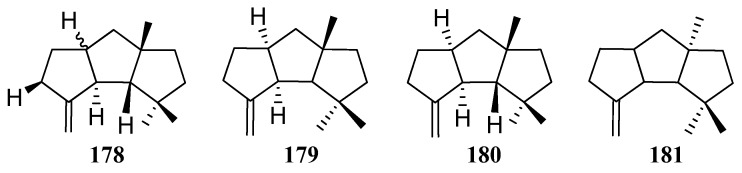
Structures of compounds **178**–**181**.

Capnellene is one of the most popular targets for synthesis, mainly owing to its molecular architecture, exocyclic olefinic linkage, disposition of methyl groups, and its role in the defense mechanism and biosynthesis of oxygenated capnellenes [75], especially (±)-∆^9(12)^-capnellene (**178**), ∆^9(12)^-capnellene (**179**), (−)-∆^9(12)^-capnellene (**180**), and (+)-∆^9(12)^-capnellene (**181**) (Figure 8). The first total synthesis of (±)-∆^9(12)^-capnellene (**178**), was reported in 1981 [76].

**Scheme 27:**
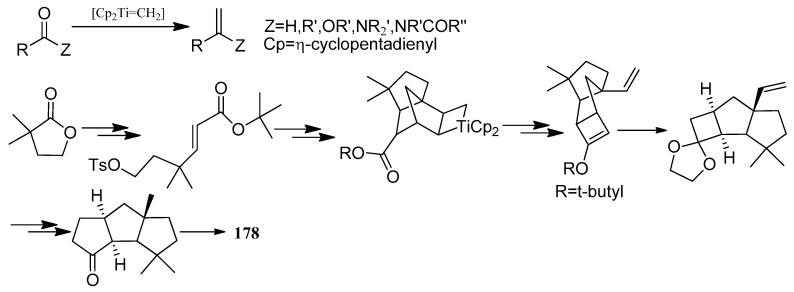
The synthesis of (±)-∆^9(12)^-capnellene (**178**) by using titanium reagents.

**Scheme 28:**
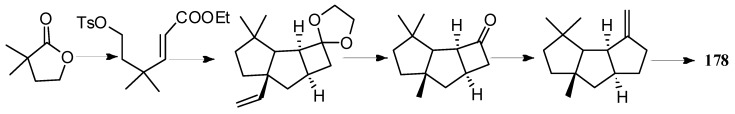
The total synthesis of **178** through the rearrangement of bicyclo[2.2.1]heptane ring systems.

**Scheme 29:**
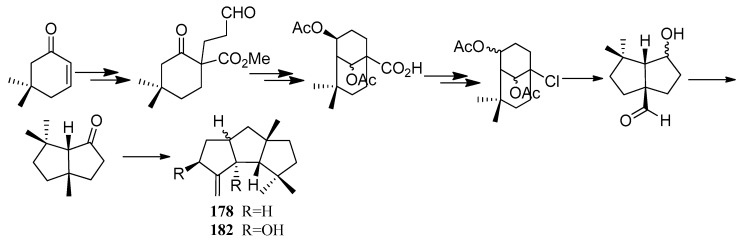
The total synthesis of both **178** and **182**.

Stille and Grubbs [77] realized the synthesis of (±)-∆^9(12)^-capnellene (**178**) by using titanium reagents (Scheme 27). Stille and co-workers [78] achieved the total synthesis of **178**, through the rearrangement of bicyclo[2.2.1]heptane ring systems by titanocene alkylidene complexes to bicyclo[3.2.0]heptane enol ethers (Scheme 28). A new synthetic strategy for (±)-∆^9(12)^-capnellene (**178**) and (±)-∆^9(12)^-capnellene-8β,10α-diol (**182**), was presented in 1992 [79]. This strategy was presented as a model study in the synthesis of the pentalanic trimethylketone, which has been used as a key intermediate in the total synthesis of both **178** and **182** (Scheme 29).

**Scheme 30:**
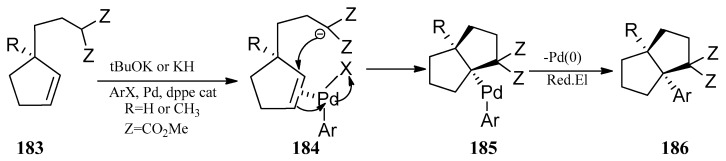
The synthesis of **186** from a new palladium catalyzed cyclisation.

**Scheme 31:**
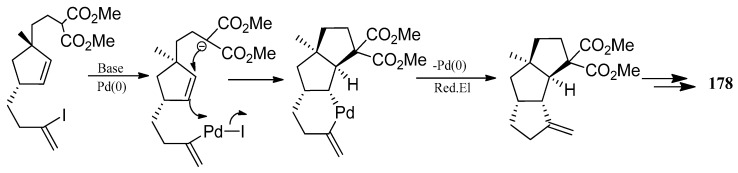
The total synthesis of **178** using a palladium-catalyzed bis-cyclization step.

It was worth mentioning that although palladium catalyzed zipper mode cyclization, using Heck reaction conditions had caused widespread concern for the construction of polycyclic system, this methodology was not suitable for the required *cis*-*anti*-*cis* stereochemistry of the capnellane system [61]. However, in 1994 Genevieve Balme achieved the total synthesis of **178**, using a palladium-catalyzed bis-cyclization step [80]. Functionalized diquinanes were easily and stereoselectively gained from a new palladium catalyzed cyclisation **183**→**186** (Scheme 30). Applying the intramolecular version of this strategy, a triquinane should be directly obtained in a single step, by construction of the two outer rings around the central ring (Scheme 31).

**Scheme 32:**
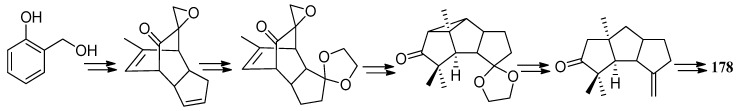
The total synthesis of **178** from p-cresol employing π^4s^ + π^2s^ cycloaddition.

In 1997, Singh and co-workers [81] developed a novel synthesis of capnellene from p-cresol and cyclopentadiene, the key steps of the synthesis were inverse demand Diels-Alder reaction and oxa-di-π-methane rearrangement [74]. They also developed a novel and efficient total synthesis of **178** from p-cresol employing π^4s^ + π^2s^ cycloaddition of spiroepoxycyclohexa-2,4-dienone and photochemical 1,2-acyl shift, as key features (Scheme 32).

**Scheme 33:**
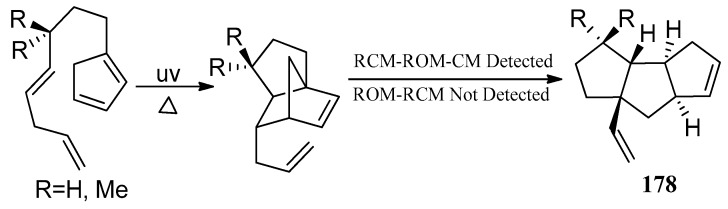
The formal synthesis of **178** via an intramolecular Diels-Alder reaction tandem metathesis protocol.

In 2009, Nguyen and co-workers [82] presented a general route to a formal synthesis of **178**. This cascade strategy, combined an intramolecular Diels-Alder reaction tandem metathesis protocol to produce the linear *cis*-*anti*-*cis*-tricyclo[6.3.0.0^2,6^]undecane skeleton directly (Scheme 33).

**Scheme 34:**
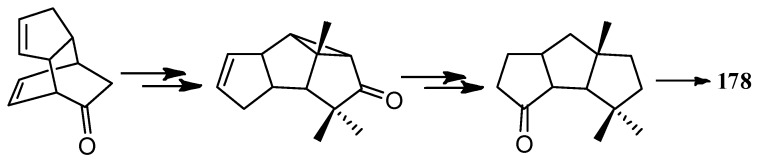
The total synthesis of **178** by using the strategy of masked *o*-benzoquinone chemistry.

In 2009, Hsu and co-workers [83] developed an efficient and short route from 2-methoxyphenols to polyfunctionalized linear triquinanes, which was characterized by the photochemistry of tricyclo[5.2.2.0^2,6^]undeca-4,10-dien-8-ones. They successfully applied this methodology to the total synthesis of **178**, in nine steps, with 20% overall yield by using the strategy of masked *o*-benzoquinone chemistry (Scheme 34).

**Scheme 35:**
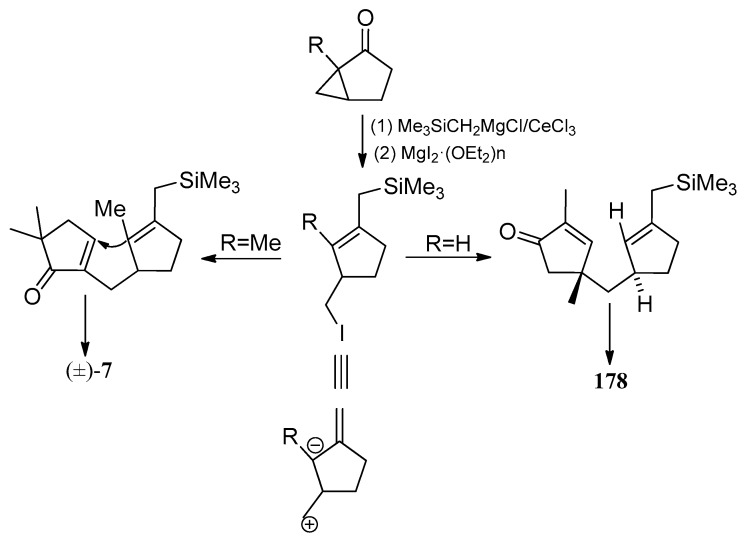
The total syntheses of (±)-**7** and **178** via a formal [3 + 2] annulation strategy.

In 2013, Shen and Li [84] achieved dual total syntheses of (±)-hirsutene ((±)-**7**) and (±)-capnellene (**178**), via a formal [3 + 2] annulation strategy. In this synthesis, the key bifunctional synthons were the cyclic homoiodo allylsilanes, which were readily prepared from the corresponding cyclopropanated cyclopentenones (Scheme 35).

**Scheme 36:**
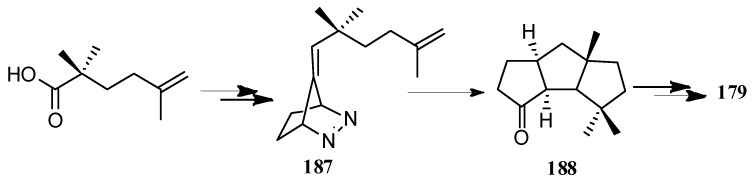
The total synthesis of ∆^9(12)^-capnellene (**179**) by using an intramolecular 1, 3-diyl trapping reaction as a primary strategy.

In 1983, a total synthesis of ∆^9(12)^-capnellene (**179**) was described by Little and co-workers [85]. They used an intramolecular 1,3-diyl trapping reaction as a primary strategy, to achieve this objective. That ketone **188** corresponded to the desired *cis, anti, cis* ring-fused product and was confirmed unambiguously by converting it to **179** with a simple Wittig reaction (Scheme 36).

**Scheme 37:**
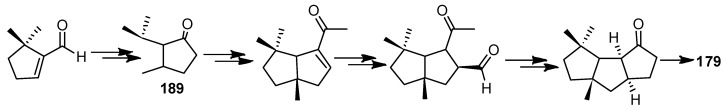
The stereocontrolled total synthesis of **179**.

In 1984, a stereocontrolled total synthesis of **179**, in 8 steps, from bicyclic enone (**189**) in 8.3% overall yield was reported (Scheme 37) [86].

**Scheme 38:**
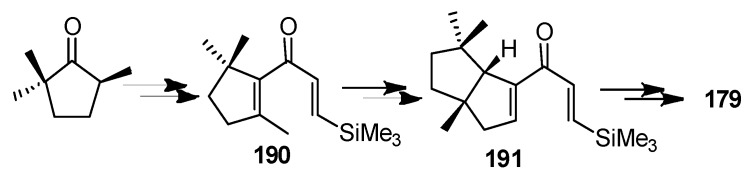
The total synthesis of **179** via palladium-catalyzed carbonylative coupling of vinyl triflates with organostannanes.

Crisp and co-workers [87] developed a method for palladium-catalyzed carbonylative coupling of vinyl triflates with organostannanes, and this methodology was applied to the total synthesis of the **179**. The approach to **179** was based on two intermediates, **190** and **191**, which were envisioned as arising from a carbonylative coupling of a vinyl triflate with vinylstannane (Scheme 38).

**Scheme 39:**
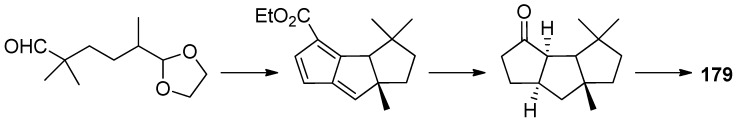
The formal synthesis of **179** by using new type of fulvenes undergo cycloadditions regioselectively.

New type of fulvenes undergo cycloadditions regioselectively to produce synthetically useful tricyclopentanoids, and a formal synthesis of **179** was reported by Ying Wang and co-workwers (Scheme 39) [88].

**Scheme 40:**
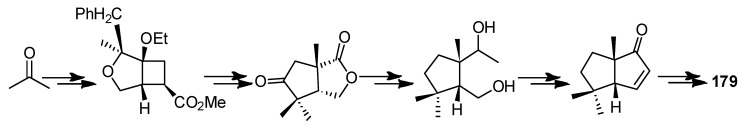
The formal synthesis of **179** via the construction of cyclopentanones with substituents up to three contiguous chiral centres.

A direct stereocontrolled route to construct cyclopentanones with substituents up to three contiguous chiral centres, had been achieved in 1998 [89], and this approach was applied in the formal synthesis of **179** (Scheme 40).

**Scheme 41:**
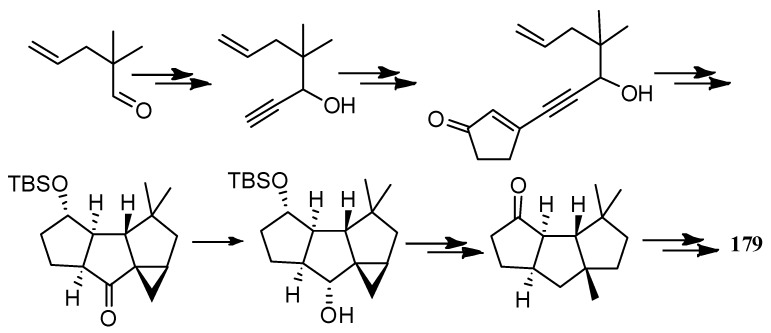
The synthesis of **179**.

In 2009, Lemiere found that cyclopentenylidene gold complexes could be easily formed from vinyl allenes, through a Nazarovlike mechanism. At the same time, polycyclic frameworks may be converted from such carbenes in four different ways [90]: Electrophilic cyclopropanation, C-H insertion, C-C migration, or proton shift. They have studied the selectivity of these different pathways and used their findings to synthesize **179** (Scheme 41).

**Scheme 42:**
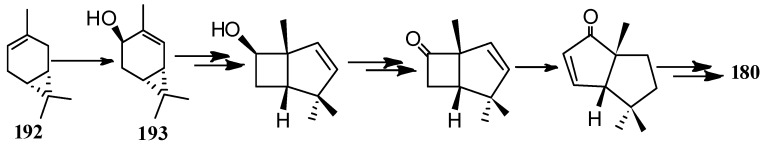
The formal asymmetric synthesis of (−)-capnellene (**180**).

A formal asymmetric synthesis of (−)-capnellene (**180**) was achieved in 1991, based on the photoinduced vinyl-cyclopropane−cyclopentene rearrangement of the bicyclic alcohol **193**, which was obtained from (+)-3-carene (**192**) (Scheme 42) [91,92].

**Scheme 43:**
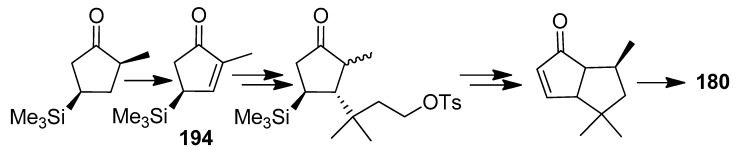
The formal synthesis of **180** by employing silyl group directed stereo control.

In 1994, Asaoka and co-workers [93] achieved a formal synthesis of **180** by employing silyl group directed stereo control, starting from (−)-2-methyl-4-trimetylsilyl-2-cyclopenten-l-one (**194**) (Scheme 43). They also noted that compound **194**, could be an advanced intermediate in the synthesis of more complex triquinanes.

**Scheme 44:**
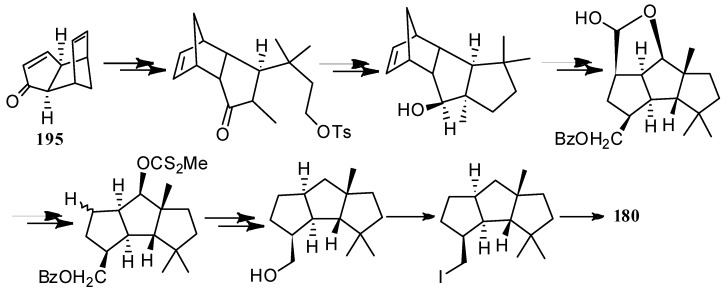
The stereocontrolled synthesis of **180** based on the structure and high functionality of the starting material.

In 1996, Tanaka and Ogasawara reported [94] the first stereocontrolled synthesis of **180**, starting from (−)-oxodicyclopentadiene (**195**). This synthesis was based on the structure and high functionality of the starting material, which resulted in the stereoselective construction of the requisite stereogenic centres on the triquinane framework easily (Scheme 44).

**Scheme 45:**
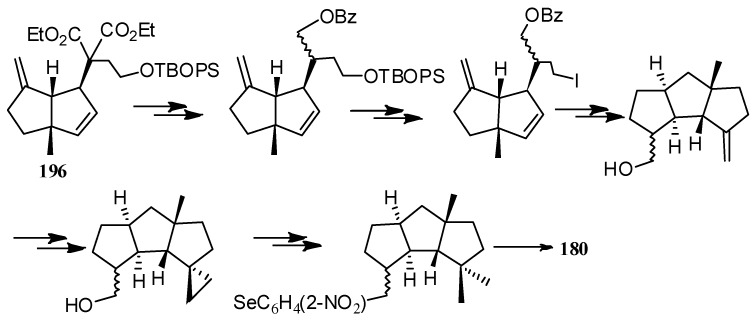
The first catalytic asymmetric total synthesis of **180** by using **196** as an asymmetric building block.

The first asymmetric Heck reaction-carbanion capture process was accomplished in 1996, by Ohshima et al. [95]. They achieved the first catalytic asymmetric total synthesis of **180**, in 19 steps, and in 20% overall yield, by using **196** as an asymmetric building block (Scheme 45).

**Scheme 46:**
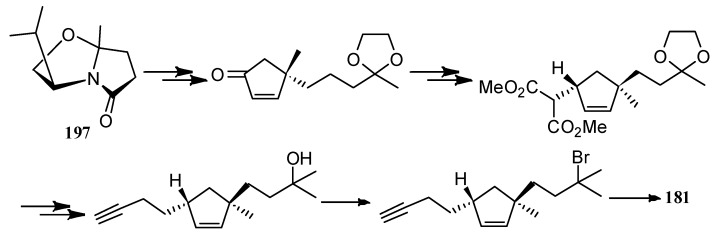
The first asymmetric total synthesis of the unnatural enantiomer of (+)-∆^9(12)^-capnellene **(181**).

The first asymmetric total synthesis of the unnatural enantiomer of (+)-∆^9(12)^-capnellene (**181**) was achieved by Meyers and Bienz, on the basis of the chiral nonracemic bicyclic lactam **197** derived from inexpensive (*S*)-valinol and levulinic acid (Scheme 46) [96].

**Scheme 47:**
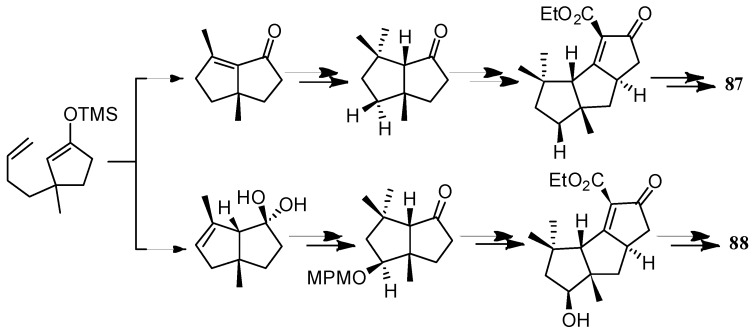
The first total syntheses of ∆^9(12)^-capnellene-8β,10α-diol (**87**) and ∆^9(12)^-capnellene-3β,8β, 10α-troil (**88**).

The first total syntheses of ∆^9(12)^-capnellene-8β,10α-diol (**87**) and ∆^9(12)^-capnellene-3β,8β, 10α-troil (**88**), was achieved by Shibasaki and co-workers [97] (Scheme 47).

**Scheme 48:**
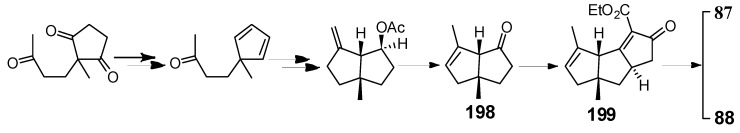
The synthesis of **87** and **88**.

Kagechika and Shibasaki [98] accomplished a catalytic asymmetric synthesis of the key intermediates **198** and **199**, for the capnellenols by an asymmetric Heck reaction, followed by an anion capture process. Furthermore, an improved synthetic route to (+)-capnellenols was developed, which led to the synthesis of ∆^9(12)^-capnellene-8β,10α-diol (**87**) and ∆^9(12)^-capnellene-3β,8β,10α-troil (**88**) (Scheme 48).

### 4.3. Other Type

**Scheme 49:**
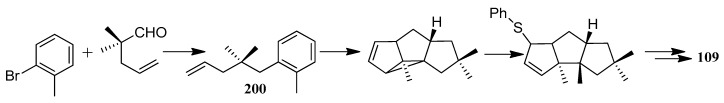
The total synthesis of racemic (±)-ceratopicanol ((±)-**109**) via intramolecular meta photocycloaddition.

**Scheme 50:**
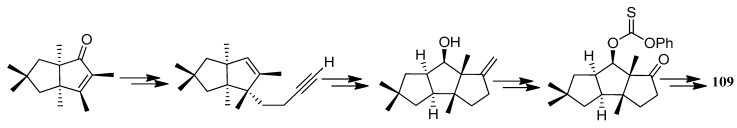
The synthesis of **109**.

In 1996, Baralotto and co-workers [99] proposed a total synthesis of racemic (±)-ceratopicanol ((±)-**109**), employing seven steps, in which the key one was an intramolecular meta photocycloaddition of the phenylpentene derivative **200**, which constituted a typical holosynthon (Scheme 49). In 1996, Clive and co-workers [100] also reported a method to synthesize (+)-ceratopicanol (**109**) (Scheme 50). This method has been used to form many compounds of the triquinane class.

**Scheme 51:**
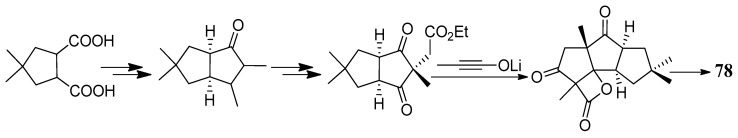
The first total synthesis of cucumin E (**78**) by using an ynolate-initiated tandem reaction for the construction of the cyclopentenone unit.

The first total synthesis of cucumin E (**78**) was achieved in 2002, which used an ynolate-initiated tandem reaction for the construction of the cyclopentenone unit (Scheme 51). This strategy proved the feasibility of a short access to triquinane sesquiterpenoids [45].

**Scheme 52:**
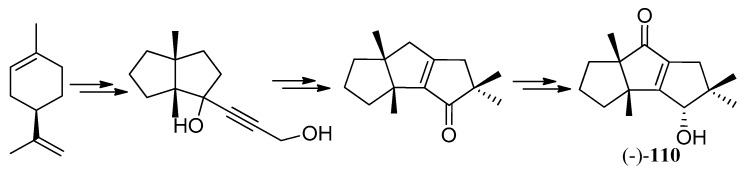
The first total synthesis of the (−)-cucumin H ((−)-**110**).

The first total synthesis of the (−)-cucumin H ((−)-**110**) was reported in 2003 [101] (Scheme 52). This method started from (R)-limonene employing two different cyclopentannulation methodologies, which were to confirm the structure, as well as establish the absolute configuration of the natural product.

**Scheme 53:**
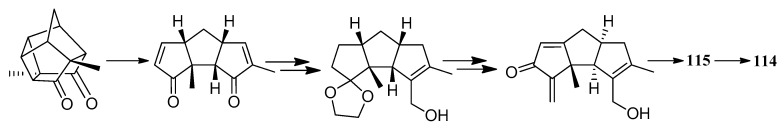
The first total synthesis of pleurotellic acid (**115**) and pleurotellol (**114**).

Furthermore in 2003, the first total synthesis of pleurotellic acid (**115**) and pleurotellol (**114**) was achieved. One notable feature of this synthesis, was that it followed an adaptation of the versatile photo-thermal metathesis-based approach to triquinane sesquiterpenoids. The other feature was the installation of the sensitive functionalization pattern in the two five-membered rings of the triquinane system, through simple reaction sequences [46] (Scheme 53).

## 5. Conclusions

From 1947 to July 2018, more than 100 linear triquinane sesquiterpenoids have been isolated, and most of them were isolated from fungi, sponges, and soft corals, among which the mushroom *stereum hirsutum*, the soft coral *capnella imbricata,* and marine fungus *chondrostereum* sp*.* were typical sources. It is important to note in particular, that Li and co-workers have obtained 19 linear triquinane sesquiterpenoids including chondrosterins A–E, I–O, hirsutanols A, C, E, F, incarnal, arthrosporone, and anhydroarthrosporone, from the marine-derived fungus *Chondrostereum* sp. Furthermore, the production of secondary metabolites often correlated with a specific stage of morphological differentiation [102]. It indicated that in addition to looking for new sources to produce linear triquinane sesquiterpenoids, we can also find ways to isolate linear triquinane sesquiterpenoids with multiple structural types from one “talented” organism. Many of these compounds displayed a variety of biological activities, such as cytotoxicity, anti-microbial, and anti-inflammatory activities. Given their structural diversity and complexity, and significant biological activities, linear triquinane sesquiterpenoids attracted great interest from synthetic chemists. Among the 8 skeletal types, type I and III are the most popular ones, a number of efficient synthesis methods have been developed to construct the unique skeleton of linear triquinane sesquiterpenoids.

## Figures and Tables

**Table 1:** Cytotoxicity of linear triquinane sesquiterpenoids.

Cell Lines	Cytotocicity
CNE1	**15** IC_50_ = 34.13 μM, **22** IC_50_ = 10.08 μM, **48** IC_50_ = 33.55 μM, **49** IC_50_ = 42.00 μM, **84** IC_50_ = 1.32 μM, **85** IC_50_ = 17.66 μM
CNE2	**15** IC_50_ = 24.87 μM, **22** IC_50_ = 12.72 μM, **43** IC_50_ = 4.95μM, **48** IC_50_ = 22.50 μM, **49** IC_50_ = 44.08 μM, **84** IC_50_ = 0.56 μM, **85** IC_50_ = 12.03 μM
HONE1	**22** IC_50_ = 17.40 μM, **48** IC_50_ = 34.60 μM, **49** IC_50_ = 46.11 μM, **85** IC_50_ = 22.06 μM
SUNE1	**22** IC_50_ = 3.50 μM, **48** IC_50_ = 30.40 μM, **49** IC_50_ = 58.83 μM, **85** IC_50_ = 16.44 μM
A549	**22** IC_50_ = 11.96 μM, **43** IC_50_ = 2.45μM, **48** IC_50_ = 29.67 μM, **49** IC_50_ = 49.58 μM, **85** IC_50_ = 23.51 μM
GLC82	**22** IC_50_ = 10.11 μM, **48** IC_50_ = 37.47 μM, **49** IC_50_ = 55.90 μM, **85** IC_50_ = 18.08 μM
HL7702	**22** IC_50_ = 9.76 μM, **48** IC_50_ = 34.26 μM, **49** IC_50_ = 56.40 μM, **85** IC_50_ = 22.14 μM
K562	**52** IC_50_ = 12.97 μg/mL, **53** IC_50_ = 16.29 μg/mL, **54** IC_50_ = 6.93 μg/mL, **55** IC_50_ = 30.52 μg/mL, **87** IC_50_ = 0.7 μM, **96** IC_50_ = 4.6 μM, **97** IC_50_ = 24 μM, **98** GI_50_ = 126.9 ± 3.0 μm/L, **99** GI_50_ = 126.9 ± 2.0 μm/L, **100** GI_50_ = 142.0 ± 4.7 μm/L, **101** GI_50_ = 142.0 ± 4.7 μm/L, Doxorubicin GI_50_ = 1.0 ± 0.6 μm/L
HCT116	**52** IC_50_ = 10.74 μg/mL, **53** IC_50_ = 16.35 μg/mL, **54** IC_50_ = 25.43 μg/mL, **55** IC_50_ = 24.17 μg/mL
HL-60	**87** IC_50_ = 51 μM, **96** IC_50_ = 68 μM, **97** IC_50_ = 713 μM
G402	**87** IC_50_ = 42–51 μM, **96** IC_50_ > 4500 μM, **97** IC_50_ = 52 μM
MCF-7	**87** IC_50_ = 93 μM, **96** IC_50_ > 4500 μM, **97** IC_50_ = 1029 μM
HT115	**87** IC_50_ = 63 μM, **96** IC_50_ > 4500 μM
A2780	**87** IC_50_ = 9.7 μM, **96** IC_50_ = 6.6 μM, **97** IC_50_ = 32 μM,
HeLa	**98** CC_50_ = 126.9 ± 1.8 μm/L, **99** CC_50_ = 126.9 ± 2.5μm/L, **100** CC_50_ = 142.0 ± 2.7 μm/L, **101** CC_50_ = 125.0 ± 2.0 μm/L, Doxorubicin CC_50_ = 2.0 ± 0.8 μm/L
L-929	**17** IC_50_ = 2.4 μg/mL, **18** IC_50_ = 0.9 μg/mL, **98** GI_50_ = 126.9 ± 2.7 μm/L, **99** GI_50_ = 126.9 ± 2.9 μm/L, **100** GI_50_ = 126.4 ± 3.0 μm/L, **101** GI_50_ = 99.1 ± 1.8 μm/L, Doxorubicin GI_50_ = 1.2 ± 0.6 μm/L
T-47D	**3** IC_50_ = 0.7 μM
SNB-75	**3** IC_50_ = 0.5 μM
HepG2	**40** IC_50_ = 24.41 ± 1.86 μM
LoVo	**43** IC_50_ = 5.47 μM

**Table 2:** Antimicrobial activity of compounds **17** and **18** (MIC_50_, μg/mL).

Test Organisms	17	18
*Bacillus cereus* DSM 318	2–5	>100
*Staphylococcus aureus* ATCC 13709	10–25	>100
*Escherichia coli* ATCC 9637	>100	>100
*Salmonella gallinarum* ATCC 9184	50–100	50–100
*Mycobacterium smegmatis* ATCC 607	25–50	50–100
*Candida albicans* ATCC 10231	50–100	>100
*Candida tropicalis* DSM 1346	>100	>100
*Rhodotorula glutinis* DSM 70398	>100	>100
*Aspergillus niger* (spores) DSM 737	1–2	25–50
*Aspergillus flavus* (spores) BD 27	2–5	25–50
*Mucor rouxii* (spores) DSM 1691	2–5	25–50

**Table 3:** Inhibitory effect of compounds **22**, **43**–**44**, **69**–**70** and **76** in LPS-induced NO production in microglial BV-2 cells.

Compounds (20 μM)	NO (μM)
**22**	8 ± 2
**43**	4 ± 1
**44**	4 ± 1
**69**	8 ± 2
**70**	10 ± 2
**76**	4 ± 1

**Table 4:** Effect of compounds **22**, **43**–**44**, **69**–**70**, and **76** in LPS-induced NO production in microglial viability.

Compounds	Viability (%)			
	2 μM	5 μM	10 μM	20 μM
**22**	94 ± 1	87 ± 3	77 ± 8	39 ± 5
**43**	nt *	nt	22 ± 3	12 ± 1
**44**	nt	nt	23 ± 7	11 ± 1
**69**	93 ± 3	82 ± 5	73 ± 4	67 ± 3
**70**	95 ± 5	95 ± 4	89 ± 9	69 ± 5
**76**	47 ± 6	30 ± 5	11 ± 2	11 ± 2

* nt = not tested.
